# Proteomic markers of Alzheimer's disease and related dementia risk in individuals with a history of depression

**DOI:** 10.1002/alz.086142

**Published:** 2025-01-09

**Authors:** Breno S Diniz, Zhiduo Chen, George A Kuchel, Richard Fortinski, Chia‐Ling Kuo

**Affiliations:** ^1^ University of Connecticut Health Center, Frmington, CT USA; ^2^ University of Connecticut Health Center, Farmington, CT USA; ^3^ University of Connecticut Medical Center, West Hartford, CT USA

## Abstract

**Background:**

History of depression is one of the most significant, and potentially modifiable risk factors for Alzheimer’s disease and related dementia (ADRD). However, there is limited evidence on the underlying biological mechanisms that link the history of depression to the higher risk of ADRD. This study aimed to evaluate the proteomic markers associated with the higher risk of ADRD.

**Method:**

We used the UK Biobank (UKB study) to address the study aim. Our sample consisted of 42,807 individuals (3,615 with a history of depression in the baseline assessment) who had plasma proteomic data available. We evaluate the proteomic predictors of the risk of ADRD among individuals with a history of depression using Cox Proportional regression and random forest analysis. The total follow‐up time was 13 years from the baseline analysis. We used the Benjamin‐Hochberg method to control for a false discovery rate.

**Result:**

A small panel of 6 proteins was significantly associated with an increased risk of progressing to ADRD upon follow‐up after controlling for age, educational level, medical comorbidity, and APOE genotype (GFAP, NFL, PSG1, GET3, HPGDS, and VGF; FDR adjusted p‐value <0.05). The predictive power of this proteomic panel was moderate, with C‐statistics = 0.79. Random forest machine learning analysis yielded similar results as the Cox regression analysis.

**Conclusion:**

This study indicates that the risk of ADRD among individuals with a history of depression is partially mediated by abnormal astroglial activation, axonal dysfunction, and neurodegeneration. Other biological mediators of risk also involve abnormalities in synaptic plastic, cell growth, peroxisomal lipid metabolism, and oxidative stress control. Our results point to novel putative mechanisms to develop interventions to mitigate the risk of ADRD among individuals with a history of depression.

